# Identifying and characterizing hepatitis C virus hotspots in Massachusetts: a spatial epidemiological approach

**DOI:** 10.1186/s12879-017-2400-2

**Published:** 2017-04-20

**Authors:** Thomas J. Stopka, Michael A. Goulart, David J. Meyers, Marga Hutcheson, Kerri Barton, Shauna Onofrey, Daniel Church, Ashley Donahue, Kenneth K. H. Chui

**Affiliations:** 1 Department of Public Health and Community Medicine, Tufts University School of Medicine, 136 Harrison Avenue, Boston, MA 02111 USA; 2 Department of Health Policy and Management, Harvard T. H. Chan School of Public Health, 677 Huntington Ave, Boston, MA 02115 USA; 3 Bureau of Infectious Disease and Laboratory Sciences, Massachusetts Department of Public Health, 350 South Street, Jamaica Plain, MA 02130 USA

**Keywords:** Getis-Ord Gi*, Spatial clusters, GIS, Surveillance, Infectious diseases

## Abstract

**Background:**

Hepatitis C virus (HCV) infections have increased during the past decade but little is known about geographic clustering patterns.

**Methods:**

We used a unique analytical approach, combining geographic information systems (GIS), spatial epidemiology, and statistical modeling to identify and characterize HCV hotspots, statistically significant clusters of census tracts with elevated HCV counts and rates. We compiled sociodemographic and HCV surveillance data (*n* = 99,780 cases) for Massachusetts census tracts (*n* = 1464) from 2002 to 2013. We used a five-step spatial epidemiological approach, calculating incremental spatial autocorrelations and Getis-Ord Gi* statistics to identify clusters. We conducted logistic regression analyses to determine factors associated with the HCV hotspots.

**Results:**

We identified nine HCV clusters, with the largest in Boston, New Bedford/Fall River, Worcester, and Springfield (*p* < 0.05). In multivariable analyses, we found that HCV hotspots were independently and positively associated with the percent of the population that was Hispanic (adjusted odds ratio [AOR]: 1.07; 95% confidence interval [CI]: 1.04, 1.09) and the percent of households receiving food stamps (AOR: 1.83; 95% CI: 1.22, 2.74). HCV hotspots were independently and negatively associated with the percent of the population that were high school graduates or higher (AOR: 0.91; 95% CI: 0.89, 0.93) and the percent of the population in the “other” race/ethnicity category (AOR: 0.88; 95% CI: 0.85, 0.91).

**Conclusion:**

We identified locations where HCV clusters were a concern, and where enhanced HCV prevention, treatment, and care can help combat the HCV epidemic in Massachusetts. GIS, spatial epidemiological and statistical analyses provided a rigorous approach to identify hotspot clusters of disease, which can inform public health policy and intervention targeting. Further studies that incorporate spatiotemporal cluster analyses, Bayesian spatial and geostatistical models, spatially weighted regression analyses, and assessment of associations between HCV clustering and the built environment are needed to expand upon our combined spatial epidemiological and statistical methods.

## Background

Approximately 71.1 million people are estimated to be currently infected with the hepatitis C virus (HCV) worldwide [1]. The number of people infected in the U.S. alone is estimated to be more than three million, of whom 75% were born between 1945 and 1965 [2]. Age-adjusted HCV mortality rates have increased steadily in recent decades [3], and notable increases in HCV cases have been reported in many states across the U.S. [4] At least 60% of prevalent HCV infections and nearly all incident HCV infections are attributed to injection drug use (IDU) [5–8], with an increasing burden among young people who inject drugs (PWID) [3–6] in non-urban, white communities [4].

Recent HCV surveillance data in Massachusetts (MA) identified a new HCV epidemic pattern characterized by a bimodal age distribution, with the expected peak among 45–65 year olds and an emerging peak among 15–30 year olds [9, 10]. There was a 78% increase in HCV cases among adolescents and young adults (15–24 years) in MA between 2002 and 2009, and an increase of 137% in the 15–29-year-old population between 2002 and 2013 [9, 10]. HCV surveillance is greatly underfunded across the United States [11], and limited spatial epidemiological and geostatistical analysis of surveillance data has been performed to date.

Innovative analytical approaches can foster a better understanding of disease transmission and improved preparation for targeted responses. Studies have demonstrated that GIS-guided approaches to disease screenings have a higher yield than traditional screening methods [12–15]. Information on where infections cluster may enhance disease prevention, treatment, and care. For example, in Connecticut, researchers used a mix of statistical and geographic information system (GIS) methods to identify unique spatial distributions and characteristics for HCV and related infections [16]. Along the U.S.-Mexico border, spatial epidemiological methods were used to identify HIV clusters, and to determine that most seroconversions were occurring in a 2.5 block radius in the red light district of Tijuana [17]. In MA, we identified HCV and HIV all-cause mortality hotspots from 2002 to 2012 [18]. However, GIS and spatial analyses to date have been relatively simplistic in their approach and new, more methodologically rigorous spatial cluster analyses are needed to pinpoint locations (i.e., “hotspots”) with highest needs for intervention. The objectives of our study were to: (1) identify HCV clusters in MA using GIS and spatial epidemiology, and (2) to characterize the HCV clusters through statistical modeling.

## Methods

We defined a hotspot as a location with a statistically significant cluster of census tracts with higher counts and rates of HCV than the average count/rate for all census tracts in MA [18].

### Data and measures

We obtained a partially de-identified, limited HCV surveillance dataset from the Massachusetts Department of Public Health (MDPH) that included 99,780 records of people with evidence of past or present infection with HCV reported from 2002 to 2013. All laboratory test results indicative of HCV infection are reportable to MDPH. Most of these reports are received via electronic laboratory reporting into the MA Virtual Epidemiologic Network (MAVEN), MDPH’s secure, web-based, electronic surveillance system [19]. The data are comprised of all newly reported probable and confirmed cases of HCV during the time period and offer the most complete window to prevalent HCV infection patterns available. CDC case definitions were employed. A probable HCV case was a case that did not meet the case definition for acute HCV, was anti-HCV positive (repeat reactive) by enzyme immunoassays (EIA), and had alanine aminotransferase (ALT or SGPT) values above the upper limit of normal, but the anti-HCV EIA result had not been verified by an additional more specific assay or the signal to cut-off ratio was unknown. A confirmed HCV case was defined as a case that was laboratory confirmed and did not meet the case definition for acute HCV. Reported variables include year of report, sex, mode of transmission (IDU vs. non-IDU), race/ethnicity, and address. Address information was missing for 15,541 records. Our final analytical dataset included 84,255 HCV cases.

#### Demographic and risk variables and rate computation

We obtained population denominators and sociodemographic data on the census tract level from U.S. Census Bureau’s American Community Survey (ACS) [20]. As of the 2010 Census, there were 1478 census tracts in MA. Using GIS and ACS data, we calculated HCV counts and rates of infection per 100,000 persons at the census tract level. Independent variables were selected based on prior research and the scientific literature. Continuous explanatory variables included race/ethnicity, age, education level, households receiving food stamps, and housing status. We used rank order histograms to determine median or tertile cut-offs based on the skewness of the data. We categorized continuous variables using the median as a cut point for non-linear increases or decreases that were monotonic. We categorized continuous variables by tertile for non-linear trends that demonstrated a U-shape or inverted U-shape. Explanatory variables for total population, and household income were dichotomized at the median, and the continuous variables for households living in poverty, households with one worker, and households with two or more workers were categorized by tertiles. Census tracts with a total population of <1 were excluded from all analyses, resulting in omission of 14 tracts (*n* = 1464).

#### Other potentially associated geographic features

Addresses for health facilities, including HIV testing sites and syringe exchanges, were obtained from MDPH. Addresses for pharmacies and prisons were obtained from the MA State Board of Pharmacy and MassGIS, respectively. Adult bookstores and gay bars were obtained through publicly available webpages and Internet directories targeting this community. All health resources and other venues that could be associated with hotspots, based on the literature and prior research, were aggregated to the census tract level.

### Spatial analyses

We used a combination of GIS, spatial epidemiological, and statistical analyses to: (1) identify the geolocation of statistically significant hotspot clusters of HCV cases, and (2) determine community-level factors (i.e., social determinants) associated with these clusters.

#### Data cleaning

We standardized address fields in SAS (v9.4, Cary, NC). The process involved parsing the addresses into different segments, unifying acronyms (e.g., changing “Str,” “Street,” “street,” and “st” into “St”), removing superfluous characters (e.g., the “#” sign), and correcting mismatched ZIP codes. The resultant segments were then rearranged into three variables: street address, town, and ZIP code, which were used for geocoding.

#### Geocoding

Geocoding is the process of obtaining longitude and latitude coordinates for an address or the geocentroid of a polygon (e.g., census tract) [21]. We geocoded the cleaned HCV cases in ArcGIS 10.2.2, using an address locator created from the U.S. Census Bureau’s 2014 Topologically Integrated Geographic Encoding and Referencing (TIGER) Line Shapefiles. We ran addresses that did not initially match through Google Earth’s desktop geocoder, which uses multiple algorithms to improve geographic match rates. Throughout our extensive cleaning processes, 66,023 observations were successfully matched in ArcMap with 16,133 more matching in Google Earth. This resulted in a total of 82,211 matched addresses for an overall match rate of 82.3% (82,211/99,780). For our analytical dataset, which included complete addresses for all HCV cases (*n* = 84,255), we achieved a match rate of 97.6% (82,211/84,255). These match rates are similar to those in other studies in the literature [22].

#### Descriptive mapping and cluster analyses

We conducted GIS and spatial epidemiological analyses to determine the burden of disease across MA. First, we created thematic GIS maps to determine the initial spatial distribution of HCV cases and rates. Data were aggregated at the census tract level to protect the confidentiality of people living with HCV while maintaining the optimal spatial resolution. Next, we used Kernel Density Estimation (KDE) to construct a smoothed surface of HCV cases across MA. KDE analyses incorporate the number and proximity of cases within defined geographic areas [23]. The final results from this method can be interpreted as the density of HCV cases per square mile.

We then used tests of spatial autocorrelation and hotspot analyses to identify the location of clusters of HCV case counts and rates per 100,000 population. Using ArcGIS 10.2.2 (ESRI, Redlands, CA), we first used incremental spatial autocorrelation at 30 different distances to determine the distance at which clustering was most intense (i.e., had the highest z-score) for each specified outcome [24]. The spatial scale (i.e., the distance) obtained from these results was then entered as a parameter in the subsequent Getis-Ord Gi* hotspot analyses. These analyses allowed us to identify the location of statistically significant clusters of census tracts with higher (or lower) values for HCV cases and infection rates [25]. Next, we conducted spatiotemporal cluster analysis (SaTScan v9.3) to determine temporal clustering patterns in micro-communities across specific years using a space-time cluster scanning statistic [26]. This model can be run at different scanning window sizes which limit the potential size of a discovered cluster. We ran this model at a 25% scanning window which we selected a priori to represent a balance of granular level cluster detection without exposing the analysis to excessive noise. Finally, we compared results across our four geostatistical analyses to observe consistencies in conclusions. Our spatial analytical methods are described in further detail elsewhere [18, 27].

### Statistical analyses

After identifying HCV hotspot clusters, we calculated descriptive statistics (Chi-squared tests and two sample t-tests) for HCV cases with and without addresses and for matched and unmatched addresses in the geocoding process to determine whether there were differences in the HCV cases by geocode status. Next, we conducted logistic regression analyses to determine the factors associated with HCV hotspots.

Statistical analysis was performed in two stages. First, to obtain the unadjusted relationship we regressed hotspot status on each of the independent variables. Variables with an overall *p*-value <0.25 were retained for the multivariable models. For multiple logistic regression analyses, significant collinearities (variance inflation factor > 6) were observed between “% of owner-occupied housing units” and “% of renter-occupied housing units”; between “% African American” and “% White; and “% Asian” and “% other race/ethnicity”; and between “% male” and “% female”. To alleviate the inflation in variance caused by the aforementioned collinearities, and based on strength of association with the outcome, we removed percentages of female, total population of owner-occupied housing, White, and Asian from the adjusted model. DFBeta and residual analyses revealed no influential points or potential outliers. A Hosmer and Lemeshow Goodness-of-Fit test was conducted with *p* = 0.46, indicating the model fit the data well. All statistical analyses were performed using SAS v9.4. Statistical significance was determined a priori at *p* < 0.05.

## Results

### Spatial analysis results

**Fig. 1:**
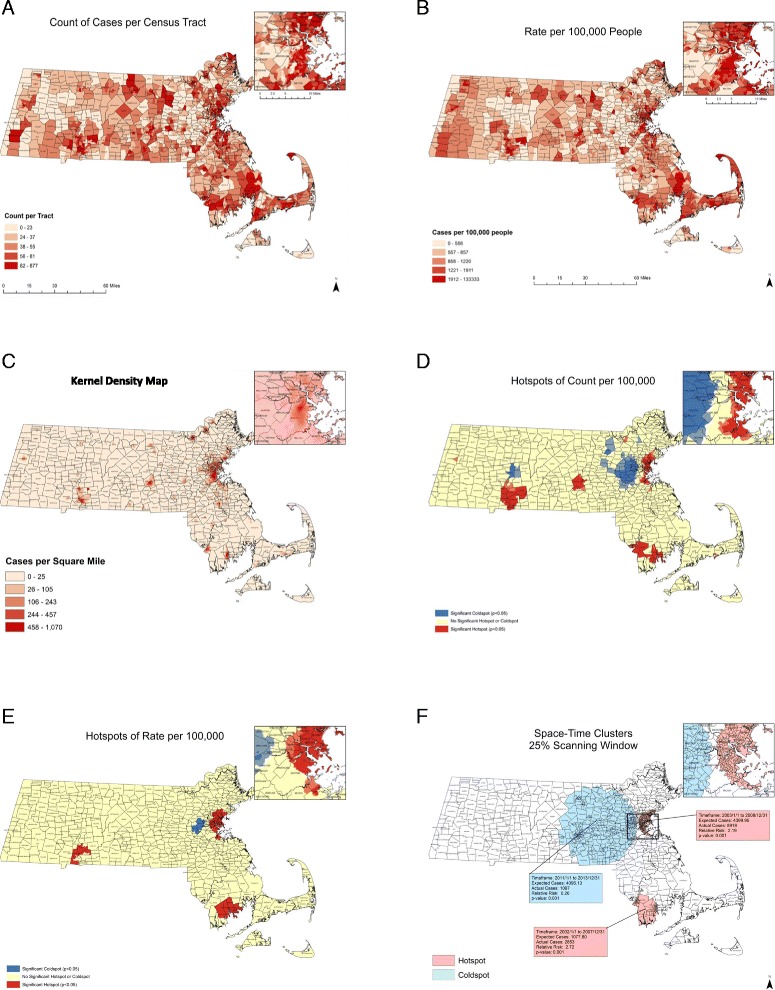
**a** HCV cases aggregated at the census tract level in Massachusetts, 2002–2013. **b** HCV rates per 100,000 in Massachusetts census tracts, 2002–2013. **c** Density of HCV cases per square mile in Massachusetts, 2002–2013. **d** HCV clusters in Massachusetts based on reported HCV counts aggregated at the census tract level (*n* = 1464). Census tracts with elevated numbers of HCV cases (red) represent hotspots (*p* < 0.05); census tracts with low numbers of HCV cases (blue) represent coldspots (*p* < 0.05); census tracts with average numbers of HCV cases are represented in yellow. **e** HCV clusters in Massachusetts based on rates per 100,000 population at the census tract level. Census tracts with elevated HCV rates (red) represent hotspots (*p* < 0.05); census tracts with low HCV rates (blue) represent coldspots (*p* < 0.05); census tracts with average HCV rates are represented in yellow; **f** Space-time clusters of HCV in Massachusetts, 2002–2013

Through initial descriptive GIS maps, we found that reported HCV infection case counts and rates were prominent across many MA municipalities between 2002 and 2013 (Fig. 1a, b). Through KDE, we identified areas with the highest densities of HCV cases per square mile. The Greater Boston area, New Bedford, Fall River, Lawrence, Lowell, Worcester, Springfield, Holyoke, and Fitchburg had densities of 477 to 1070 HCV cases per square mile (Fig. 1c). We identified nine statistically significant hotspot clusters for HCV case *counts*, with the largest clusters in Boston, New Bedford/Fall River, Worcester, and Springfield (*p* < 0.05) (Fig. 1d). We found clusters for HCV *rates* per 100,000 persons in Boston, New Bedford, and Springfield (Fig. 1e). Through space-time cluster analyses, we detected similar geographic clusters, with a large coldspot in the Metro West region, and hotspots in the Greater Boston area and the South Shore of Massachusetts (Fig. 1f). We ran hotspot cluster analyses separately for HCV cases among 15–30 year olds and 45–65 year olds and no notable differences in spatial clustering patterns were detected. In Table 1, we highlight agreement in results across our four geostatistical analytical methods, and rank areas of highest concern.

**Table 1:** Comparison of geostatistical findings highlighting locations with high and low reported HCV infections, Massachusetts, 2002–2013

Location	Kernel density analysis	Getis-Ord GI* hotspot test (count)	Getis-Ord GI* hotspot test (rate)	Poisson space-time cluster test
Greater Boston area	Higher caseload per square mile	Statistically significant hot spot	Statistically significant hot spot	Statistically significant hot spot
South shore of Massachusetts	Higher caseload per square mile	Statistically significant hot spot	Statistically significant hot spot	Statistically significant hot spot
Springfield	Higher caseload per square mile	Statistically significant hot spot	Statistically significant hot spot	No significant cluster
Worcester	Higher caseload per square mile	Statistically significant hot spot	No significant cluster	No significant cluster
Pittsfield	Higher caseload per square mile	Statistically significant hot spot	No significant cluster	No significant cluster
Cape Cod	Higher caseload per square mile	Statistically significant hot spot	No significant cluster	No significant cluster
North shore of Massachusetts	Higher caseload per square mile	No significant cluster	No significant cluster	No significant cluster
Merrimack Valley	Higher caseload per square mile	No significant cluster	No significant cluster	No significant cluster
Metro West	No discernable difference from state average	Statistically significant cold spot	Statistically significant cold spot	Statistically significant cold spot

### Statistical results

In Table 2, we present descriptive statistics for the characteristics of the HCV cases we analyzed, comparing HCV cases with and without addresses, and HCV cases with addresses that were successfully and unsuccessfully matched through geocoding. We noted significant differences across HCV case characteristics in both comparisons.

**Table 2:** Descriptive statistics of HCV cases by address availability and geocoding match status

	Address			Geocoding		
	Missing	Present	*p*-value	Unmatched	Matched	*p*-value
**Count**	14,980 (15.01%)	84,800 (84.99%)		2589 (3.05%)	82,211 (96.95%)	
**Mean Age**	43.5	42.1	<0.001	42.3028	42.09298	0.462
**Gender %**			<0.001			0.004
Male	56.69	61.26		64.39	61.16	
Female	29.93	37.29		34.26	37.38	
Other/Unknown	13.38	1.46		1.35	1.46	
**Exposure %**			<0.001			<0.001
PWID	8.72	24.88		27.54	24.79	
Non-PWID	1.59	5.62		4.56	5.65	
Unknown	89.69	69.51		67.9	69.56	
**Year %**			<0.001			<0.001
2002	13.62	8.17		9.08	8.14	
2003	7.92	7.83		8.88	7.8	
2004	7.32	9.32		16.92	9.08	
2005	6.04	7.56		14.99	7.33	
2006	7.52	7.95		7.69	7.96	
2007	9.95	9.04		7.26	9.09	
2008	9.13	8.71		7.38	8.75	
2009	9.81	7.99		5.45	8.07	
2010	8.5	7.73		4.98	7.82	
2011	8.22	8.48		6.26	8.55	
2012	6.96	8.84		5.79	8.93	
2013	5.01	8.38		5.33	8.47	

All *p*-values are the result of chi-squared tests with the exception of Mean age which is a result of a two sample t-test

We present descriptive statistics for HCV hotspot and non-hotspot census tracts in Table 3. We noted significant differences, across race/ethnicity, gender, and socioeconomic variables. In bivariate logistic regression analyses (Table 4), total population, population density, percent of population that was Hispanic, percent high school graduates or higher, percent living in poverty, median household income, and median age were associated with HCV hotspots. In multivariable logistic regression analyses, we found that HCV hotspots were independently and positively associated with the percent of the population that was Hispanic (adjusted odds ratio [AOR]: 1.07; 95% confidence interval [CI]: 1.04, 1.09) and the percent of households receiving food stamps (AOR: 1.83; 95% CI: 1.22, 2.74). HCV hotspots were independently and negatively associated with the percent of the population that were high school graduates or higher (AOR: 0.91; 95% CI: 0.89, 0.93) and the percent of the population in the “other” race/ethnicity category (AOR: 0.88; 95% CI: 0.85, 0.91) (Table 4).

**Table 3:** Descriptive statistics of Massachusetts census tracts, 2002–2013 (*n* = 1464)

Characteristic	Census tracts in HCV hotspot (n = 302)Mean (95% CI)	Census tracts outside HCV hotspot (n = 1162)Mean (95% CI)
**Demographic Variables**		
Male^a^, %	48.32 (47.99, 48.48)	48.23 (47.99, 48.48)
Total population, no. per census tract	3837 (3663, 4010)	4577 (4481, 4673)
Median age, years	34.94 (34.20, 35.68)	39.71 (39.32, 40.09)
High School graduate or higher^b^, %	81.59 (80.03, 83.15)	89.18 (88.61, 89.75)
White^a, c^, %	70.57 (68.14, 73.00)	82.99 (81.83, 84.14)
African American^a, c^, %	10.44 (8.88, 11.99)	6.25 (5.49, 7.01)
Hispanic^a, d,^, %	16.80 (14.74, 18.86)	8.33 (7.50, 9.16)
Asian^a, c^, %	7.60 (6.51, 8.69)	4.49 (4.14, 4.84)
Other Race or Ethnicity^a, e^, %	7.56 (6.43, 8.69)	4.35 (3.79, 4.91)
**Socioeconomic Variables**		
Median household income, $	53,066 (50,543, 55,590)	71,725 (70,006, 73,444)
Households receiving food stamps^a, g^, %,	13.86 (12.36, 15.37)	8.07 (7.48, 8.66)
Households living at poverty status^a, g^, (%)	10.16 (9.17, 11.15)	12.40 (11.73, 13.08)
Households with 1 worker^a, g^, (%)	27.85 (26.95, 28.75)	29.26 (28.71, 29.80)
Households with ≥2workers^a,g^, (%)	59.53 (58.22, 60.83)	58.07 (57.33, 58.82)
Owner-occupied housing units, %	42.78 (40.22, 45.34)	67.46 (66.10, 68.82)
Renter-occupied housing units, %	57.22 (54.66, 59.78)	32.54 (31.18, 33.90)
**Structural Variables**		
Pharmacies, no.	1.14 (0.93, 1.35)	1.50 (1.38, 1.62)
Gay bars, no.	0.04 (0.02, 0.07)	0.02 (0.00, 0.05)
Adult bookstores, no.	0.02 (0.01, 0.04)	0.02 (0.01, 0.03)
Syringe exchanges, no.	0.007 (0.00, 0.02)	0.00 (0.00, 0.00)
HIV testing sites, no.	0.36 (0.22, 0.49)	0.10 (0.07, 0.14)
Prisons, no.	0.02 (0.00, 0.03)	0.03 (0.02, 0.05)
HCV cases^f^, no.	72.31 (65.87, 78.74)	51.96 (49.33, 54.90)
HCV rate per 100,000 individuals^f^, no.	2424 (1544, 3303)	1220 (1154, 1286)

*n* sample size, *%* percent, *$* United States dollar, *HCV* hepatitis C virus, *HIV* human immunodeficiency virus, *CI* confidence interval, *IQR* interquartile range

^a^Percent of total population within census tract

^b^Population 25 years of age or older

^c^Non-Hispanic

^d^Of any race

^e^Includes Native Hawaiian/Pacific Islander, American Indian/Alaskan Native, and other races/ethnicities not specified

^f^Reported HCV cases to the Massachusetts Department of Public Health from 2002 to 2013

^g^Last 12 months

**Table 4:** Factors associated with HCV hotspots in Massachusetts, 2002–2013

Characteristic		Unadjusted model OR (95% CI)	Adjusted model^†^ AOR (95% CI)
Male^a^	< 48.25%	*Referent*	
	≥ 48.25%	0.88 (0.68, 1.14)	--
Female^a^	< 51.75%	*Referent*	
	≥ 51.75%	1.13 (0.88, 1.46)	--
Total population, No. per census tract	< 4248	*Referent*	*Referent*
	≥ 4248	*0.50 (0.38, 0.64)*	1.08 (0.80, 1.47)
Median age, years		*0.90 (0.89, 0.92)*	1.02 (0.99, 1.06)
High School graduate or higher^b^, %		*0.95 (0.94, 0.96)*	*0.91 (0.89, 0.93)*
White^a, c^, %		*0.98 (0.97, 0.98)*	--
African American^a,c^, %		*1.02 (1.01, 1.03)*	1.01 (1.00, 1.02)
Hispanic^a,d,^, %		*1.03 (1.02, 1.04)*	*1.07 (1.04, 1.09)*
Asian^a, c^, %		*1.05 (1.04, 1.07)*	--
Other Race or Ethnicity^a, e^, %		*1.03 (1.02, 1.04)*	0.88 (0.85, 0.91)
Median annual household income, $	< $65,571	*Referent*	*Referent*
	≥ $65,571	*0.32 (0.24, 0.42)*	0.91 (0.60, 1.39)
Households receiving food stamps^f^, %	< 4.85%	*Referent*	*Referent*
	≥ 4.85%	*3.01 (2.29, 3.95)*	*1.83 (1.22, 2.74)*
Households living in poverty^f^	< 5.6%	*Referent*	*Referent*
	≥ 5.6% to <11.8%	1.01 (0.73, 1.37)	1.19 (0.81, 1.73)
	≥ 11.8%	0.73 (0.53, 1.00)	1.38 (0.91, 2.08)
Households with one worker^f^	< 24.6%	*Referent*	*Referent*
	≥ 24.6% to <31.0%	0.77 (0.56, 1.04)	1.28 (0.84, 1.95)
	≥ 31.0%	0.71 (0.51, 0.97)	1.37 (0.79, 2.36)
Households with two or more workers^f^	< 55.6%	*Referent*	*Referent*
	≥ 55.6% to <64.7%	1.01 (0.73, 1.39)	0.80 (0.51, 1.27)
	≥ 64.7%	1.27 (0.94, 1.73)	1.06 (0.60, 1.90)
Renter-occupied housing units, %		*1.04 (1.03, 1.05)*	1.00 (0.99, 1.01)
Pharmacies, no.		*0.91 (0.85, 0.97)*	0.96 (0.90, 1.04)
Gay bars, no.		1.07 (0.85, 1.35)	--
Adult bookstores, no.		1.06 (0.50, 2.26)	--
Syringe exchanges, no.		3.87 (0.54, 27.56)	1.61 (0.18, 14.59)
HIV testing sites, no.		*1.37 (1.19, 1.57)*	0.97 (0.81, 1.160)
Prisons, no.		0.67 (0.31, 1.44)	--

*n* sample size, *%* percent, *$* United States dollar, *HCV* hepatitis C virus, *HIV* human immunodeficiency virus, *SD* standard deviation, *IQR* interquartile range, *OR* odds ratio, *AOR* adjusted odds ratio, *CI* confidence interval. Italicized text represents statistically significant results (*p* < 0.05)

^†^Adjusted for: number of pharmacies, number of syringe exchanges, number of HIV testing sites, percent of the total population that was a high school graduate or higher, total population, median household income, percent of renter-occupied housing units, percent of households received food stamps, percent of total population that was Hispanic, percent of total population that was African American, percent of total population that was another race/ethnicity, percent of households living at poverty status, percent of households with one worker, and percent of households with two or more workers

^a^Percent of total population within census tract

^b^Population 25 years of age or older

^c^Non-Hispanic

^d^Of any race

^e^Includes Native Hawaiian/Pacific Islander, American Indian/Alaskan Native, and other races/ethnicities not specified

^f^Last 12 months

## Discussion

Our combined use of GIS, spatial epidemiological, and statistical modeling approaches allowed us to identify and characterize statistically significant geographic HCV hotspot clusters. We observed agreement across the different geostatistical methods we conducted, which lends support to the idea that these locations in MA present significantly greater burdens of disease. Our HCV hotspots based on HCV counts highlighted nine clusters, the largest of which included Boston, Fall River, New Bedford, Worcester, and Springfield, where the burden of disease is highest. We detected hotspots for HCV rates per 100,000 persons in Boston, New Bedford, and Springfield. Given that these hotspots are based on rates, controlling for population differences across census tracts in MA, they may represent locations with some of the greatest need for enhanced HCV prevention and treatment interventions. It is notable that each of these cities has a population with lower median age than those found across MA, and that authorized syringe exchange programs have not been available in New Bedford and Springfield, despite initiation of such programs elsewhere in MA in the 1990s, and Worcester, Lawrence and a number of additional sites in 2016.

In our multivariable analyses, we found that the percent of population that was Hispanic and the percent of households receiving food stamps were independently and positively associated with HCV hotspots, while the percent of the population that was another race/ethnicity and the percent of the population that had a high school education or higher were negatively associated with HCV hotspots. These associations help us to understand the underlying sociodemographic factors that are associated with HCV clusters. The associations we observed with racial/ethnic groups differ from recent findings that highlight an emerging HCV epidemic among non-urban, white youth and younger populations [4]. However, the associations we observed between high school education, belonging to the “other” racial/ethnic groups, and HCV hotspots echo the significant health disparities that exist among different racial/ethnic minorities, educational attainment, and income levels for HCV [28]. Since the surveillance data upon which our analyses depend cover the timeframe from 2002 to 2013, it may not be possible to discern more recent patterns in disease transmission. Confirmed and probable cases of HCV infection include individuals that have past exposure, but not current infection. Analysis of more recent surveillance data may begin to uncover different patterns, including the emerging epidemic among young, suburban, white communities.

Our findings may have implications for future disease transmission risks and for the future success of HCV treatment among PWID, for which there are many sound recommendations [29]. PWID have experienced a number of barriers along the HCV care cascade, which includes diagnosis of disease, linkage to care, treatment, retention, and cure. Targeting of future treatment or cure as prevention (CasP) approaches [30, 31] could benefit from spatial epidemiological analyses that identify and characterize HCV hotspot clusters. Antiviral treatment uptake among PWID has been limited by barriers at patient, provider, and structural levels [32]. With the advent of direct acting antiviral (DAA) therapy, many payers, including most state Medicaid programs, continue to require abstinence from illicit drug use and often restrict treatment by fibrosis stage [33]. These policies systematically exclude many PWID who have not yet suffered from decades of infection and present barriers to performing real-life CasP interventions in locations and populations with the highest risk of HCV transmission. In MA, as of August 2016, all versions of Medicaid and most private insurers approve DAAs at fibrosis stage F0 or higher and without strict sobriety criteria, facilitating PWID treatment. However, multiple barriers remain for PWID along the cascade, resulting in a very small fraction likely to reach cure [31].

There are several limitations in the current study. First, HCV surveillance systems may not capture all HCV cases. While MDPH’s MAVEN surveillance and electronic laboratory reporting system has led to improved efficiency and timeliness in capturing HCV data [19], disease surveillance data only include information on individuals who have been tested and reported to health authorities. People who are not tested or are not engaged in care are not represented in these data. Race/ethnicity data are missing or incomplete for many HCV cases, making it difficult to discern representation in the HCV surveillance data by race/ethnicity. Second, the MAVEN system captures address at time of report, typically many years after initial HCV infection, and residence rather than place of infection. As a result, spatial results may represent risk for HCV transmission 10–20 years earlier. However, in the absence of large cohort studies that follow PWID over time, starting at a young age and allowing for calculation of HCV incidence rates and a better understanding of the geographic location where transmission occurred, such case reporting systems offer the best option currently available on a statewide level. Third, our dataset includes people who are positive for anti-HCV antibody through EIA. It is possible that some HCV cases tested positive for antibody but negative for HCV ribonucleic acid, which would over-estimate HCV cases of prevalent infection. Spontaneous viral clearance occurs in approximately 25% of people initially diagnosed with HCV [34]. However, it is unlikely that there are differential spatial distributions for reported HCV cases that ultimately clear the virus compared to cases that do not. Fourth, in our statistical models, we assessed associations at the census tract level, rather than the individual level. HCV surveillance (outcomes) and ACS data (explanatory variables) for the entire state are included in our analyses, and represent the best data available on the micro (i.e., neighborhood) level, and have been used in previous small area analyses with similar outcomes [35]. Finally, differences in characteristics of HCV cases with and without addresses and for matched and unmatched addresses following geocoding can help to illustrate which groups and characteristics may be underrepresented and can inform training needs for completion of future HCV case report forms, which can further enhance the precision of HCV surveillance data as well as spatial and statistical analyses.

### Future research

There are a number of unique spatial analytical tools and approaches that are available to explore HCV, and other infection clustering patterns, in MA and the US. Limited spatial epidemiological and geostatistical analysis of surveillance data has been performed to date, and the spatial uncertainty of such analyses is unknown. Future research should incorporate spatiotemporal cluster analyses, additional spatial modeling approaches, including Bayesian spatial and geostatistical models, and spatially weighted regression analyses, to enhance spatial precision and build upon our approaches. In addition, nuanced analyses that compare clustering patterns across different population subgroups and the role of the built environment are needed to determine differences by age cohort, risk behaviors, environment, and race/ethnicity over time.

## Conclusions

Combined geospatial and statistical analyses can help identify regions of risk and regions of disease, and inform public health policy decisions. Through our analytical approach, we identified and characterized clusters in MA where enhanced HCV screening, prevention, treatment, and care could help to combat the HCV epidemic.

## Abbreviations

ACS: American Community Survey
AOR: Adjusted Odds Ratio
CDC: Centers for Disease Control and Prevention
CI: Confidence Interval
EIA: Enzyme Immunoassay
GIS: Geographic Information System
HCV: Hepatitis C Virus
HIV: Human Immunodeficiency Virus
KDE: Kernel Density Estimates
MA: Massachusetts
MAVEN: Massachusetts Virtual Epidemiologic Network
MDPH: Massachusetts Department of Public Health
PWID: People who Inject Drugs
TIGER: Topologically Integrated Geographic Encoding and Referencing
